# 5-(1*H*-Imidazol-1-ylsulfon­yl)-*N*,*N*-dimethyl­naphthalen-1-amine

**DOI:** 10.1107/S160053680804066X

**Published:** 2008-12-10

**Authors:** Lei Teng, Yong Zhang, Shi-lei Zhang, Yuan Qu, Xian-you Xia

**Affiliations:** a School of Chemical and Materials Engineering, Huangshi Institute of Technology, Huangshi 435003, People’s Republic of China

## Abstract

In the title mol­ecule, C_15_H_15_N_3_O_2_S, the dihedral angle between the naphthalene ring system and the imidazole ring is 86.1 (2)°. In the crystal structure, weak inter­molecular C—H⋯O and C—H⋯N hydrogen bonds, as well as weak C—H⋯π inter­actions, connect mol­ecules, forming a two-dimensional network.

## Related literature

For background information, see: Corradini *et al.* (1997 ▶); Kavallieratos *et al.* (2005 ▶); Koike *et al.* (1996 ▶). For the synthesis, see: Hilderbrand *et al.* (2004 ▶). 
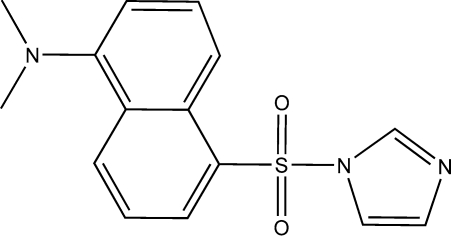


## Experimental

### 

#### Crystal data

C_15_H_15_N_3_O_2_S
*M* *_r_* = 301.36
Orthorhombic, 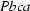

*a* = 16.3707 (16) Å
*b* = 7.7928 (7) Å
*c* = 22.088 (2) Å
*V* = 2817.8 (5) Å^3^
*Z* = 8
Mo *K*α radiation
μ = 0.24 mm^−1^
*T* = 150 (2) K
0.20 × 0.10 × 0.10 mm

#### Data collection

Bruker SMART CCD diffractometer
Absorption correction: multi-scan (*SADABS*; Sheldrick, 1996 ▶) *T*_min_ = 0.944, *T*_max_ = 0.977
9800 measured reflections
2577 independent reflections
2196 reflections with *I* > 2σ(*I*)
*R*_int_ = 0.058

#### Refinement

*R*[*F* ^2^ > 2σ(*F* ^2^)] = 0.041
*wR*(*F* ^2^) = 0.110
*S* = 1.07
2577 reflections
192 parameters
H-atom parameters constrained
Δρ_max_ = 0.50 e Å^−3^
Δρ_min_ = −0.31 e Å^−3^

### 

Data collection: *SMART* (Bruker, 2007 ▶); cell refinement: *SAINT-Plus* (Bruker, 2007 ▶); data reduction: *SAINT-Plus*; program(s) used to solve structure: *SHELXS97* (Sheldrick, 2008 ▶); program(s) used to refine structure: *SHELXL97* (Sheldrick, 2008 ▶); molecular graphics: *PLATON* (Spek, 2003 ▶); software used to prepare material for publication: *SHELXTL* (Sheldrick, 2008 ▶).

## Supplementary Material

Crystal structure: contains datablocks global, I. DOI: 10.1107/S160053680804066X/lh2737sup1.cif

Structure factors: contains datablocks I. DOI: 10.1107/S160053680804066X/lh2737Isup2.hkl

Additional supplementary materials:  crystallographic information; 3D view; checkCIF report

## Figures and Tables

**Table 1:** Hydrogen-bond geometry (Å, °)

*D*—H⋯*A*	*D*—H	H⋯*A*	*D*⋯*A*	*D*—H⋯*A*
C6—H6⋯O2	0.95	2.42	3.057 (2)	125
C15—H15⋯N3^i^	0.95	2.45	3.395 (3)	173
C14—H14⋯O2^ii^	0.95	2.45	3.358 (3)	161
C13—H13⋯N1^iii^	0.95	2.57	3.506 (2)	169
C10—H10⋯*Cg*^iv^	0.95	2.82	3.302 (2)	113

Symmetry codes: (i) 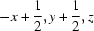; (ii) 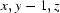; (iii) 
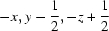
; (iv) 
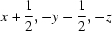
. *Cg* is the centroid of the C3–C7/C12 ring.

**Fig. 1.**
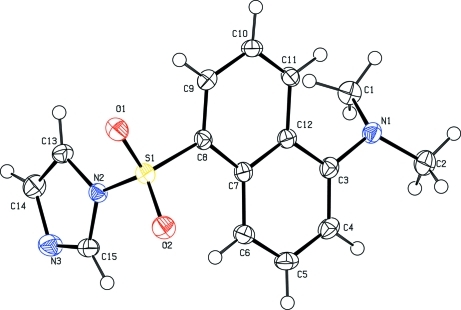
The molecular structure of (I), with displacement ellipsoids drawn at the 50% probability level.

**Fig. 2.**
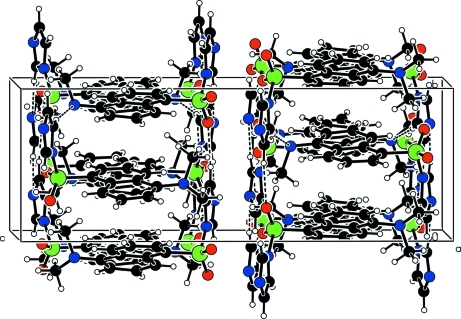
Part of the crystal structure of (I) showing weak hydrogen bonds as dashed lines.

## supplementary crystallographic information

### Comment

Dansyl chloride is widely used as a fluorescent label in immunofluorescence methods and in yielding fluorescent N-terminal amino acids and peptide derivatives. Some dansyl chloride derivatives are also used as fluorescent probes, which can detect trace metal ions such as Pb^2+^, Cu^2+^, Zn^2+^ (Koike *et al.*, 1996; Corradini *et al.*, 1997; Kavallieratos *et al.*, 2005). We are interested in preparing fluorescent drug or ligand analogs that are expected to bind to hydrophobic sites in proteins or membranes. With this mind, the title compound, (I), was prepared and we report the crystal stucture herein.

In the molecular structure (Fig. 1), the dihedral angle between the naphthalene ring and the imidazole ring is 86.1 (2)°. All bond lengths and bond angles are as expected. In the crystal structure (Fig.2), the molecules are linked by C–H···O and C—H···N hydrogen bonds (Table 1) and C—H···π interactions into a two-dimension network.

### Experimental

Compound (I) was synthesized according to a literature procedure (Hilderbrand *et al.*, 2004). Single crystals suitable for X-ray diffraction were obtained by slow evaporation of an ethyl acetate solution of (I) at room temperature.

### Refinement

All H atoms were placed in idealized positions [*C*—*H*(methyl)=0.98 Å and C—H(aromatic) =0.95 Å] and included in the refinement in the riding-model approximation, with *U*_iso_(H_methyl_)= 1.5*U*_eq_(C) and *U*_iso_(H_aromatic_) = 1.2*U*_eq_(C).

### Crystal data

**Table:**

C_15_H_15_N_3_O_2_S	*F*(000) = 1264
*M**_r_* = 301.36	*D*_x_ = 1.421 Mg m^−^^3^
Orthorhombic, *P**b**c**a*	Mo *K*α radiation, λ = 0.71073 Å
Hall symbol: -P 2ac 2ab	Cell parameters from 3852 reflections
*a* = 16.3707 (16) Å	θ = 2.2–28.2°
*b* = 7.7928 (7) Å	µ = 0.24 mm^−^^1^
*c* = 22.088 (2) Å	*T* = 150 K
*V* = 2817.8 (5) Å^3^	Block, red
*Z* = 8	0.20 × 0.10 × 0.10 mm

### Data collection

**Table:**

Bruker SMART CCD diffractometer	2577 independent reflections
Radiation source: fine-focus sealed tube	2196 reflections with *I* > 2σ(*I*)
graphite	*R*_int_ = 0.058
φ and ω scans	θ_max_ = 25.5°, θ_min_ = 1.8°
Absorption correction: multi-scan (*SADABS*; Sheldrick, 1996)	*h* = −12→19
*T*_min_ = 0.944, *T*_max_ = 0.977	*k* = −7→9
9800 measured reflections	*l* = −26→25

### Refinement

**Table:**

Refinement on *F*^2^	Primary atom site location: structure-invariant direct methods
Least-squares matrix: full	Secondary atom site location: difference Fourier map
*R*[*F*^2^ > 2σ(*F*^2^)] = 0.041	Hydrogen site location: inferred from neighbouring sites
*wR*(*F*^2^) = 0.110	H-atom parameters constrained
*S* = 1.07	*w* = 1/[σ^2^(*F*_o_^2^) + (0.0583*P*)^2^ + 0.6727*P*] where *P* = (*F*_o_^2^ + 2*F*_c_^2^)/3
2577 reflections	(Δ/σ)_max_ < 0.001
192 parameters	Δρ_max_ = 0.50 e Å^−^^3^
0 restraints	Δρ_min_ = −0.31 e Å^−^^3^

### Special details

**Table:**

Geometry. All e.s.d.'s (except the e.s.d. in the dihedral angle between two l.s. planes) are estimated using the full covariance matrix. The cell e.s.d.'s are taken into account individually in the estimation of e.s.d.'s in distances, angles and torsion angles; correlations between e.s.d.'s in cell parameters are only used when they are defined by crystal symmetry. An approximate (isotropic) treatment of cell e.s.d.'s is used for estimating e.s.d.'s involving l.s. planes.
Refinement. Refinement of *F*^2^ against ALL reflections. The weighted *R*-factor *wR* and goodness of fit *S* are based on *F*^2^, conventional *R*-factors *R* are based on *F*, with *F* set to zero for negative *F*^2^. The threshold expression of *F*^2^ > σ(*F*^2^) is used only for calculating *R*-factors(gt) *etc*. and is not relevant to the choice of reflections for refinement. *R*-factors based on *F*^2^ are statistically about twice as large as those based on *F*, and *R*- factors based on ALL data will be even larger.

### Fractional atomic coordinates and isotropic or equivalent isotropic displacement parameters (Å^2^)

**Table:**

	*x*	*y*	*z*	*U*_iso_*/*U*_eq_	
C1	0.11008 (13)	−0.0608 (3)	0.10523 (10)	0.0274 (5)	
H1A	0.1612	−0.1147	0.0922	0.041*	
H1B	0.0721	−0.0558	0.0710	0.041*	
H1C	0.0858	−0.1285	0.1380	0.041*	
C2	0.18098 (13)	0.2027 (3)	0.08508 (9)	0.0319 (5)	
H2A	0.1877	0.3221	0.0981	0.048*	
H2B	0.1574	0.2002	0.0443	0.048*	
H2C	0.2343	0.1457	0.0846	0.048*	
C3	0.14436 (11)	0.1301 (3)	0.18942 (8)	0.0216 (4)	
C4	0.21791 (12)	0.1896 (3)	0.21123 (9)	0.0247 (5)	
H4	0.2602	0.2184	0.1836	0.030*	
C5	0.23134 (11)	0.2084 (3)	0.27371 (9)	0.0255 (5)	
H5	0.2825	0.2507	0.2874	0.031*	
C6	0.17273 (11)	0.1673 (3)	0.31536 (9)	0.0234 (4)	
H6	0.1840	0.1773	0.3574	0.028*	
C7	0.09481 (11)	0.1098 (2)	0.29548 (9)	0.0193 (4)	
C8	0.02739 (11)	0.0735 (2)	0.33507 (9)	0.0193 (4)	
C9	−0.04868 (11)	0.0311 (3)	0.31351 (9)	0.0214 (4)	
H9	−0.0917	0.0063	0.3410	0.026*	
C10	−0.06277 (11)	0.0244 (3)	0.25099 (9)	0.0221 (4)	
H10	−0.1159	−0.0007	0.2360	0.026*	
C11	−0.00036 (11)	0.0539 (2)	0.21174 (9)	0.0207 (4)	
H11	−0.0107	0.0476	0.1695	0.025*	
C12	0.08007 (11)	0.0938 (2)	0.23193 (9)	0.0201 (4)	
C13	0.06482 (12)	−0.2592 (3)	0.42799 (9)	0.0235 (5)	
H13	0.0112	−0.2976	0.4187	0.028*	
C14	0.13037 (12)	−0.3564 (3)	0.44095 (9)	0.0280 (5)	
H14	0.1302	−0.4783	0.4420	0.034*	
C15	0.17381 (12)	−0.0973 (3)	0.44626 (9)	0.0238 (5)	
H15	0.2077	0.0005	0.4514	0.029*	
N1	0.12680 (9)	0.1138 (2)	0.12696 (7)	0.0224 (4)	
N2	0.09210 (9)	−0.0906 (2)	0.43108 (7)	0.0189 (4)	
N3	0.19833 (10)	−0.2548 (2)	0.45265 (8)	0.0295 (4)	
O1	−0.04379 (8)	0.04896 (19)	0.43910 (6)	0.0269 (4)	
O2	0.08176 (8)	0.22535 (18)	0.43451 (6)	0.0274 (4)	
S1	0.03535 (3)	0.08123 (6)	0.41480 (2)	0.02021 (17)	

### Atomic displacement parameters (Å^2^)

**Table:**

	*U*^11^	*U*^22^	*U*^33^	*U*^12^	*U*^13^	*U*^23^
C1	0.0290 (10)	0.0248 (13)	0.0283 (11)	0.0020 (9)	0.0010 (9)	−0.0029 (9)
C2	0.0303 (11)	0.0407 (14)	0.0248 (10)	−0.0036 (10)	0.0055 (9)	0.0055 (10)
C3	0.0222 (9)	0.0177 (11)	0.0251 (10)	0.0019 (8)	0.0009 (8)	0.0024 (8)
C4	0.0194 (9)	0.0245 (12)	0.0302 (11)	−0.0013 (8)	0.0048 (8)	0.0025 (9)
C5	0.0178 (9)	0.0250 (12)	0.0337 (11)	−0.0014 (8)	−0.0041 (8)	−0.0010 (9)
C6	0.0226 (9)	0.0224 (12)	0.0251 (10)	−0.0001 (8)	−0.0045 (8)	−0.0002 (8)
C7	0.0199 (9)	0.0163 (11)	0.0218 (10)	0.0023 (8)	−0.0009 (7)	0.0015 (8)
C8	0.0220 (9)	0.0158 (11)	0.0201 (10)	0.0016 (8)	−0.0015 (7)	0.0013 (8)
C9	0.0208 (9)	0.0197 (11)	0.0238 (10)	−0.0010 (8)	0.0026 (8)	0.0008 (8)
C10	0.0181 (9)	0.0211 (11)	0.0270 (11)	−0.0021 (8)	−0.0042 (8)	−0.0001 (9)
C11	0.0227 (10)	0.0205 (12)	0.0190 (9)	0.0003 (8)	−0.0044 (8)	0.0015 (8)
C12	0.0192 (9)	0.0164 (11)	0.0247 (11)	0.0015 (8)	−0.0011 (8)	0.0021 (8)
C13	0.0246 (10)	0.0234 (12)	0.0226 (10)	−0.0060 (9)	−0.0018 (8)	−0.0014 (8)
C14	0.0337 (11)	0.0187 (12)	0.0316 (11)	−0.0002 (9)	−0.0014 (9)	−0.0017 (9)
C15	0.0200 (9)	0.0247 (12)	0.0266 (11)	−0.0024 (8)	−0.0030 (8)	0.0002 (8)
N1	0.0231 (8)	0.0233 (10)	0.0209 (9)	−0.0022 (7)	0.0025 (7)	0.0018 (7)
N2	0.0207 (8)	0.0179 (9)	0.0182 (8)	0.0004 (6)	−0.0016 (6)	−0.0003 (6)
N3	0.0247 (9)	0.0259 (11)	0.0377 (10)	0.0024 (7)	−0.0056 (7)	0.0012 (8)
O1	0.0232 (7)	0.0357 (9)	0.0216 (7)	0.0041 (6)	0.0028 (5)	−0.0006 (6)
O2	0.0325 (8)	0.0221 (8)	0.0276 (7)	0.0006 (6)	−0.0025 (6)	−0.0041 (6)
S1	0.0221 (3)	0.0203 (3)	0.0183 (3)	0.00270 (19)	−0.00034 (18)	−0.00119 (19)

### Geometric parameters (Å, °)

**Table:**

C1—N1	1.468 (3)	C8—C9	1.374 (3)
C1—H1A	0.9800	C8—S1	1.767 (2)
C1—H1B	0.9800	C9—C10	1.401 (3)
C1—H1C	0.9800	C9—H9	0.9500
C2—N1	1.457 (2)	C10—C11	1.359 (3)
C2—H2A	0.9800	C10—H10	0.9500
C2—H2B	0.9800	C11—C12	1.424 (3)
C2—H2C	0.9800	C11—H11	0.9500
C3—C4	1.377 (3)	C13—C14	1.344 (3)
C3—N1	1.415 (2)	C13—N2	1.389 (3)
C3—C12	1.438 (3)	C13—H13	0.9500
C4—C5	1.405 (3)	C14—N3	1.390 (3)
C4—H4	0.9500	C14—H14	0.9500
C5—C6	1.367 (3)	C15—N3	1.299 (3)
C5—H5	0.9500	C15—N2	1.380 (2)
C6—C7	1.422 (3)	C15—H15	0.9500
C6—H6	0.9500	N2—S1	1.6692 (16)
C7—C12	1.430 (3)	O1—S1	1.4249 (14)
C7—C8	1.436 (3)	O2—S1	1.4241 (14)
			
N1—C1—H1A	109.5	C10—C9—H9	120.0
N1—C1—H1B	109.5	C11—C10—C9	119.90 (17)
H1A—C1—H1B	109.5	C11—C10—H10	120.0
N1—C1—H1C	109.5	C9—C10—H10	120.0
H1A—C1—H1C	109.5	C10—C11—C12	122.14 (18)
H1B—C1—H1C	109.5	C10—C11—H11	118.9
N1—C2—H2A	109.5	C12—C11—H11	118.9
N1—C2—H2B	109.5	C11—C12—C7	118.84 (17)
H2A—C2—H2B	109.5	C11—C12—C3	120.99 (17)
N1—C2—H2C	109.5	C7—C12—C3	120.00 (16)
H2A—C2—H2C	109.5	C14—C13—N2	105.42 (17)
H2B—C2—H2C	109.5	C14—C13—H13	127.3
C4—C3—N1	123.28 (17)	N2—C13—H13	127.3
C4—C3—C12	118.54 (17)	C13—C14—N3	110.95 (19)
N1—C3—C12	118.04 (16)	C13—C14—H14	124.5
C3—C4—C5	121.01 (18)	N3—C14—H14	124.5
C3—C4—H4	119.5	N3—C15—N2	111.22 (17)
C5—C4—H4	119.5	N3—C15—H15	124.4
C6—C5—C4	121.79 (18)	N2—C15—H15	124.4
C6—C5—H5	119.1	C3—N1—C2	116.91 (16)
C4—C5—H5	119.1	C3—N1—C1	116.09 (16)
C5—C6—C7	119.71 (18)	C2—N1—C1	110.30 (16)
C5—C6—H6	120.1	C15—N2—C13	106.73 (16)
C7—C6—H6	120.1	C15—N2—S1	128.48 (14)
C6—C7—C12	118.84 (17)	C13—N2—S1	124.69 (13)
C6—C7—C8	124.31 (17)	C15—N3—C14	105.70 (17)
C12—C7—C8	116.79 (16)	O2—S1—O1	120.60 (9)
C9—C8—C7	122.21 (18)	O2—S1—N2	105.66 (8)
C9—C8—S1	114.88 (14)	O1—S1—N2	106.46 (9)
C7—C8—S1	122.91 (14)	O2—S1—C8	111.78 (9)
C8—C9—C10	119.99 (17)	O1—S1—C8	107.60 (8)
C8—C9—H9	120.0	N2—S1—C8	103.20 (8)
			
N1—C3—C4—C5	178.02 (18)	N2—C13—C14—N3	0.5 (2)
C12—C3—C4—C5	2.5 (3)	C4—C3—N1—C2	−15.4 (3)
C3—C4—C5—C6	0.5 (3)	C12—C3—N1—C2	160.21 (18)
C4—C5—C6—C7	−2.3 (3)	C4—C3—N1—C1	117.6 (2)
C5—C6—C7—C12	0.9 (3)	C12—C3—N1—C1	−66.8 (2)
C5—C6—C7—C8	−176.03 (19)	N3—C15—N2—C13	0.0 (2)
C6—C7—C8—C9	174.8 (2)	N3—C15—N2—S1	−176.46 (14)
C12—C7—C8—C9	−2.2 (3)	C14—C13—N2—C15	−0.3 (2)
C6—C7—C8—S1	−4.7 (3)	C14—C13—N2—S1	176.33 (14)
C12—C7—C8—S1	178.26 (14)	N2—C15—N3—C14	0.3 (2)
C7—C8—C9—C10	−0.8 (3)	C13—C14—N3—C15	−0.5 (2)
S1—C8—C9—C10	178.69 (15)	C15—N2—S1—O2	−14.58 (19)
C8—C9—C10—C11	2.4 (3)	C13—N2—S1—O2	169.57 (15)
C9—C10—C11—C12	−0.7 (3)	C15—N2—S1—O1	−143.94 (17)
C10—C11—C12—C7	−2.5 (3)	C13—N2—S1—O1	40.22 (18)
C10—C11—C12—C3	−177.88 (19)	C15—N2—S1—C8	102.91 (18)
C6—C7—C12—C11	−173.40 (18)	C13—N2—S1—C8	−72.93 (17)
C8—C7—C12—C11	3.8 (3)	C9—C8—S1—O2	−137.71 (15)
C6—C7—C12—C3	2.1 (3)	C7—C8—S1—O2	41.82 (18)
C8—C7—C12—C3	179.25 (17)	C9—C8—S1—O1	−3.13 (18)
C4—C3—C12—C11	171.62 (19)	C7—C8—S1—O1	176.40 (16)
N1—C3—C12—C11	−4.2 (3)	C9—C8—S1—N2	109.19 (16)
C4—C3—C12—C7	−3.7 (3)	C7—C8—S1—N2	−71.28 (17)
N1—C3—C12—C7	−179.52 (17)		

### Hydrogen-bond geometry (Å, °)

**Table:**

*D*—H···*A*	*D*—H	H···*A*	*D*···*A*	*D*—H···*A*
C6—H6···O2	0.95	2.42	3.057 (2)	125
C15—H15···N3^i^	0.95	2.45	3.395 (3)	173
C14—H14···O2^ii^	0.95	2.45	3.358 (3)	161
C13—H13···N1^iii^	0.95	2.57	3.506 (2)	169
C10—H10···Cg^iv^	0.95	2.82	3.302 (2)	113

Symmetry codes: (i) −*x*+1/2, *y*+1/2, *z*; (ii) *x*, *y*−1, *z*; (iii) −*x*, *y*−1/2, −*z*+1/2; (iv) *x*+1/2, −*y*−1/2, −*z*.
